# GATAD1 gene amplification promotes glioma malignancy by directly regulating CCND1 transcription

**DOI:** 10.1002/cam4.2405

**Published:** 2019-07-08

**Authors:** Shanshan Zhang, Min Gao, Lin Yu

**Affiliations:** ^1^ Department of Radiology Tianjin Medical University General Hospital Tianjin China; ^2^ Department of Biochemistry and Molecular Biology School of Basic Medical Sciences of Tianjin Medical University Tianjin China

**Keywords:** chromatin architectural interaction, GATAD1, gene amplification, glioma, prognosis biomarker

## Abstract

**Background:**

The GATAD1 gene overexpression induced by GATAD1 amplification upregulation is detected in different human tumors. To date, the relationship between GATAD1 amplification and glioma oncogenesis and malignancy is still unknown.

**Methods:**

GATAD1 gene amplification and expression were analyzed in 187 gliomas using qPCR and immunostaining. The relation of GATAD1 to patients’ prognoses was assessed via the Kaplan–Meier method. The MTT and orthotopic tumor transplantation assays were used to identify the function of GATAD1 in glioma proliferation. cDNA microarray, ChIP qPCR, EMSA and 3C were used to screen the downstream mechanism of GATAD1 regulating glioma proliferation.

**Results:**

Our results indicated that GATAD1 gene amplification and GATAD1 gene expression are novel independent diagnosis biomarkers to indicate poor outcome of glioma patients. GATAD1 knockdown can remarkably suppress GBM cell proliferation both in vitro and in vivo. GATAD1 could promote *CCND1* gene transcription by inducing long range chromatin architectural interaction on the CCND1 promoter. Then GATAD1 sequentially accelerates GBM cell cycle transition and proliferation via regulating CCND1.

**Conclusions:**

We identify GATAD1 as a novel potential diagnosis biomarker and promising prognosis predictor in glioma patients. Functionally, we confirm GATAD1 as an epigenetic chromatin topological regulator that promotes glioma proliferation by targeting CCND1.

## 1 INTRODUCTION

Glioma is the most common primary brain tumor.^1^ Among glioma, the glioblastoma (GBM) manifests the highest lethality and incidence of recurrence.^2^, ^3^ Gene copy number aberrations, such as gene amplification/deletion and chromosome gain/loss, commonly occurr in glioma patients and are considered as the major reason and hallmark event of oncogenesis process.^4^, ^5^ The current glioma histopathological criteria by World Health Organization (WHO) also adopts the aberration as a molecular diagnosis biomarker.^6^ In glioma patients, copy number codeletion at chromosomal region 1p/19q is characteristic of oligodendrogliomas and closely associated with improved survival.^7^, ^8^ The copy number loss of chromosome 10 in different grades glioma is also reported and correlated with important oncogenes such as PTEN.^9^, ^10^, ^11^ Unfortunately, the prognosis variations among individual patients still significantly interferes with the accuracy of glioma diagnosis.^12^, ^13^ Therefore, it is very urgently needed to characterize new biomarker genes with copy number aberrations correlated with glioma diagnosis outcome. Furthermore, unraveling the oncogenic molecular mechanisms of the identified novel gene targets with copy number variation could also contribute to understand the glioma oncogenesis process and to develop better therapeutic methods to reduce mortality of patients.

The cell cycle transition is driven by different cyclins and cyclin‐dependent kinases. CCND1, also named as cyclin D1, belongs to D cyclin family and drives the cell cycle progression of G1 to S phase.^14^, ^15^ The CCND1 overexpression or CCND1 downstream pathway  aberrant activation could directly promote neoplastic growth. Therefore CCND1 overexpression could be detected in different human tumors such as hepatocellular carcinoma, breast cancer and glioma.^16^, ^17^, ^18^

In this manuscript, we identified copy number amplification of GATA zinc finger domain containg 1 (GATAD1) in glioma tissues. The GATAD1 expression level is also positively correlated with its own gene amplification condition. GATAD1 is located at chromatin 7q21.2, which is a location associated with malignancy in other tumors.^19^ However, the molecular mechanism of GATAD1 in glioma is still unclear. In this study, we characterized that, by targeting CCND1, GATAD1 gene amplification is associated with GATAD1 overexpression and glioma progression.

## 2 MATERIALS AND METHODS

### 2.1 Patients’ samples and clinical feature

One hundred and eighty seven glioma tissue samples and 20 nonmalignant brain tissue samples were obtained from Tianjin Medical University General Hospital (TMUGH). All glioma patients were officially informed and signed consent forms. Glioma samples were first fixed with formaldehyde and embedded in paraffin for immunohistochemistry (IHC) staining. The diagnoses of the above sample were given by two individual pathologists under the guidance of the WHO glioma classifcation criteria. The diagnoses results were summarized in Table S1. The TMUGH ethics committee had confirmed that all procedures were handled following the Helsinki Declaration. The TCGA GBM + LGG dataset was also used to analyze GATAD1 gene amplification and expression condition (https://cancergenome.nih.gov/).

### 2.2 Copy number assay

GATAD1 copy number was detected using TaqMan Copy Number Assays kit (assay ID: Hs02569007_cn, Hs04928809_cn, ABI). The RNaseP TaqMan Copy Number Reference was used as internal reference control (assay id: 4403326; ABI). Relative quantification (RQ) result was analyzed using Copy Caller Software (Applied Biosystems).

### 2.3 IHC

The VECTASTAIN Elite ABC HRP kit (VECTOR, Burlingame) was used to detect GATAD1 protein level in glioma samples. A GATAD1 antibody was diluted at 1:400 (Santa Cruz, Dallas, USA). The IHC images were collected using a Leica DM6000B microscope (Wetzlar, Germany). The percentage of positive staining cells among all cells was set as Labeling index (LI [%]).

### 2.4 Cell culture

U87MG cells were purchased from ATCC. U251 cells were obtained from the Cell Bank of Chinese Academy of Sciences. The cells were cultured in DMEM with 10% FBS (Gibco) at 37°C, 5% CO_2_. The mycoplasma contamination was detected using MycoProbe Mycoplasma Detection Kit (R&D) on 12 March 2019.

### 2.5 Proliferation assay

The U87MG and U251 cells were cultured in 96‐well plates (3 × 10^3^ cells/well) for 24 hours. The cells were cultured for additional 1, 2, 3, 4 and 5 days after attachment. Then culture medium containing 0.5% 3‐(dimethylthiazol‐2‐yl)‐2,5‐diphenyltetrazoliumbromide (MTT; Sigma) was loaded to every well and treated for 2 hours. The medium was replaced with dimethyl sulfoxide (150 μL per well; Sigma) to dissolve the crystals. The OD at 490 nm was measured using the Varioskan Flash Multimode Reader (Thermo Fisher).

### 2.6 ChIP‐qPCR assays

The chromatin was precipitated using GATAD1 antibody (8 μg; Santa Cruz) and IgG control antibody (8 μg; Millipore) respectively, then the DNA was extracted and detected using EZ‐Magna‐ChIP™‐Kit (Millipore) following the manufacturer's instruction. All the PCR primers were synthesized by Sangon and shown in Table S2.

### 2.7 GBM cell line orthotopic transplantation

U87MG cells stably expressed firefly luciferase via lentivirus transfection. The cells were cotransfected with lentiviruses containing scramble sequence (scramble) or GATAD1 shRNA sequences (GATAD1‐sh1: 5'‐CGGCTGCTGAAAAGAAAGTCTCCAC‐3', GATAD1‐sh2: 5'‐TGGAAAGCCCTACTATGCTCAAATC‐3'). These cells were injected (7.5 × 10^4^ cells) into non‐obese diabetic severe combined immunodeficiency (NOD‐SCID) mice brains (6‐week‐old). The tumor luminescence images were taken using IVIS Spectrum living animal image system (Perkin Elmer) after 28 days via 200 mg/g D‐luciferin injection. All animal handling procedures were conducted under the guidance of the Tianjin Medical University Institutional Animal Care and Use Committee.

### 2.8 IDH mutation detection

Genomic DNA of the glioma tissues was purified using the QIAamp DNA FFPE Tissue Kit (Qiagen). The purified genomic DNA was sent to Gene‐tech to detect IDH mutation condition using Sanger sequencing.

### 2.9 Electrophoretic mobility shift assay

The probes used in Electrophoretic mobility shift assay (EMSA) were purchased from Beyotime. All probes information have been summarized in Table S2. We used the Light Shift Chemiluminescent EMSA kit (Thermo) to perform the EMSA. The negative control mixture was contained a biotin‐labeled probe (20 fmol); the binding group was mixed with biotin probe (20 fmol) and nuclear extract (10 μg); the competition reaction contained biotin probe (20 fmol), nuclear extract (10 μg) and competitive probe (100 fmol); and the mutated competition group contained biotin probe (20 fmol), nuclear extract (10 μg) and mutated competitive probe (100 fmol). After half an hour of incubation at 37°C, all the mixtures were loaded onto 7% native PAGE gel for 45 minutes electrophoresis at 85 V.

### 2.10 qRT‐PCR

Total RNA was extracted using TRIzol reagent (Invitrogen) was reverse transcribed to cDNA using RevertAid First Strand cDNA Synthesis Kit (Fermentas) for further qPCR detection (FastStart Universal SYBR Green kit, Sigma). The PCR reactions were performed on StepOne™ Real‐Time PCR System (Applied Biosystems). The PCR procedure included an initial 30 seconds denaturation step at 95°C, followed by a 1 minute extension step at 60°C for 40 cycles. GAPDH was used as reference gene. Primers were purchased from Sangon and are summarized in Table S3.

### 2.11 Western blot

After purified by RIPA buffer (Thermo), the BCA method was used to detect the protein concentrations of each sample. Total amount of 20 μg protein was loaded for SDS‐PAGE, then the antibodies against GATAD1, CCND1 and β‐actin were used to detect corresponding proteins.

### 2.12 Chromosome conformation capture

The formaldehyde fixed purified chromatin was treated with TaqI for fragmentation and religated with T4 DNA ligase. qPCR was performed to detect the crosslink effect. The randomly ligated TaqI treated CCND1 promoter fragments were used as positive control. All the primers are listed in Table S4.

### 2.13 Statistics analyses

The statistics analysis was performed using SPSS software (IBM, Chicago, USA, version 21.0). Numeric data are represented as the mean ± SD and compared using One‐way ANOVA. The correlations were detected using Pearson correlation. The survival analysis was performed using Kaplan‐Meier (KM) method (disease‐free survival: DFS, overall survival: OS), the subgrouping of the samples were stratified by gene amplification condition or medians of gene expression levels. The survival factors were further analyzed using Cox's regression. *P* < 0.05 were set as significance; *P* < 0.05 (*), *P* < 0.01 (**), or *P* < 0.001 (***). All cell line experiments were repeated three times.

## 3 RESULTS

### 3.1 GATAD1 gene amplification indicates high glioma grades and poor prognosis

**Figure 1:**
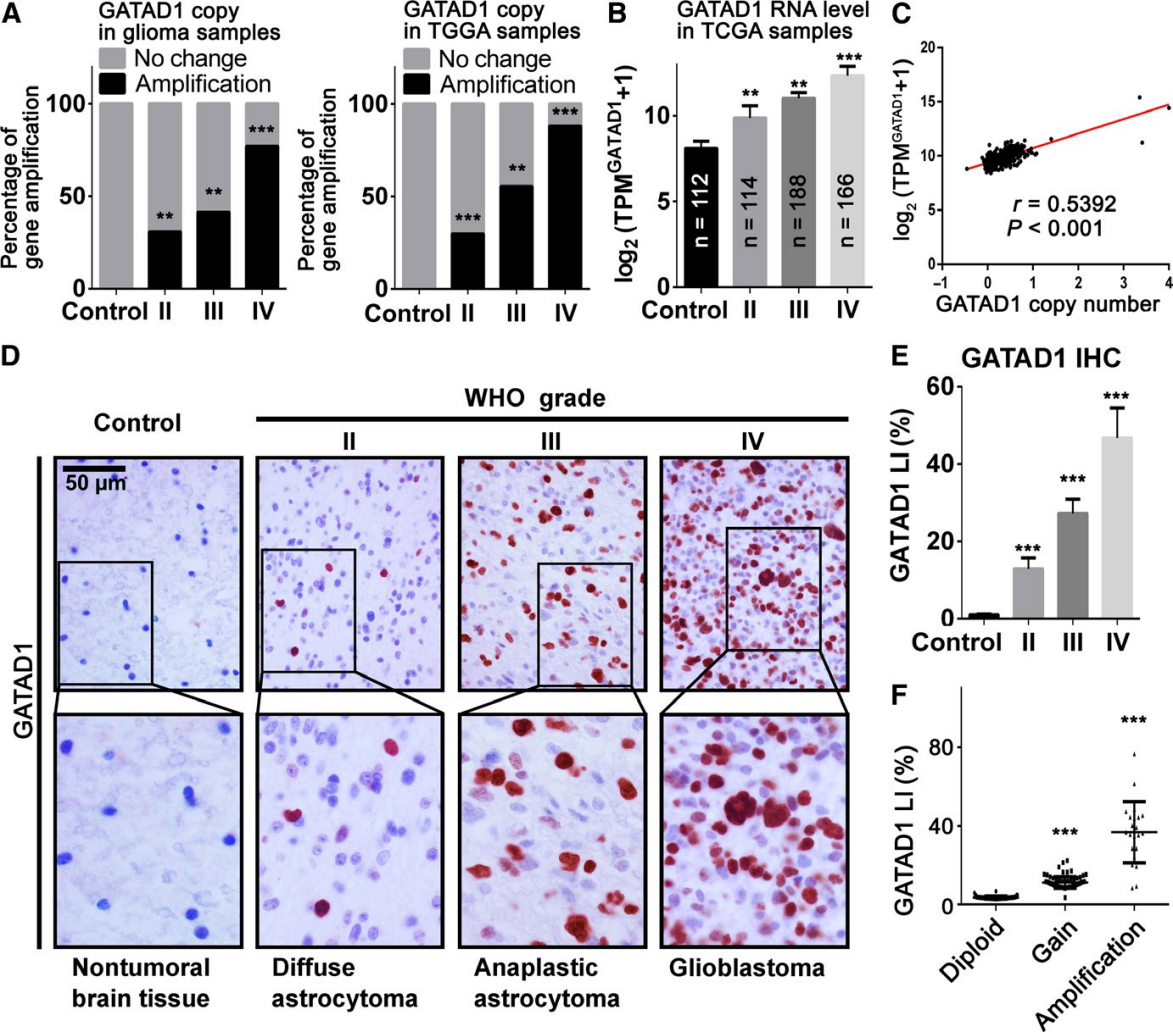
The amplification condition and expression level of GATAD1 is positively correlated to glioma grade. (A) GATAD1 gene amplification condition from our World Health Organization (WHO) grade II‐IV glioma samples (left, n = 187) and TCGA dataset (right, LGG + GBM, n = 468). (B) Comparison of GATAD1 RNA level among different grades glioma from TCGA samples. (C) GATAD1 copy number status was positively correlated with its mRNA expression in TCGA samples. (D) immunohistochemistry (IHC) staining of GATAD1 in the FFPE samples of 187 gliomas and 20 nonmalignant brain tissues; scale bar = 50 μm. (E) GATAD1 protein level Labeling index (LI [%]) is correlated with glioma grades (n = 187). (F) GATAD1 gene copy gain was identified in 56.2% (105/187) of our glioma samples. Data are presented as mean ± SD. **P < 0.01; ***P < 0.001

In our 187 glioma samples, we evaluated significant GATAD1 gene copy amplification in WHO grade II‐IV patients. More importantly, we found that the incidences of GATAD1 amplification were correlated with the glioma grade. These results were further verified using whole genome sequencing data from TCGA LGG and GBM dataset (Figure 1A). The mRNA level of GATAD1 from TCGA also correlated with the glioma grade (Figure 1B); liner regression results further showed positive correlation between mRNA level and GATAD1 copy number in TCGA dataset (Figure 1C, *r* = 0.5392, *P* < 0.001). We further detected the GATAD1 protein level in our glioma samples using IHC staining, the results indicated that GATAD1 LI (%) was increased in high‐grade glioma (Figure 1D,E). Similarly, GATAD1 protein levels were positively correlated with GATAD1 copy number (Figure 1F). The above data suggested that the GATAD1 copy number amplification contributed to hyper‐expression of GATAD1 and indicated higher WHO grade in glioma.

### 3.2 GATAD1 gene amplification indicates shorter survival times of glioma patients

**Figure 2:**
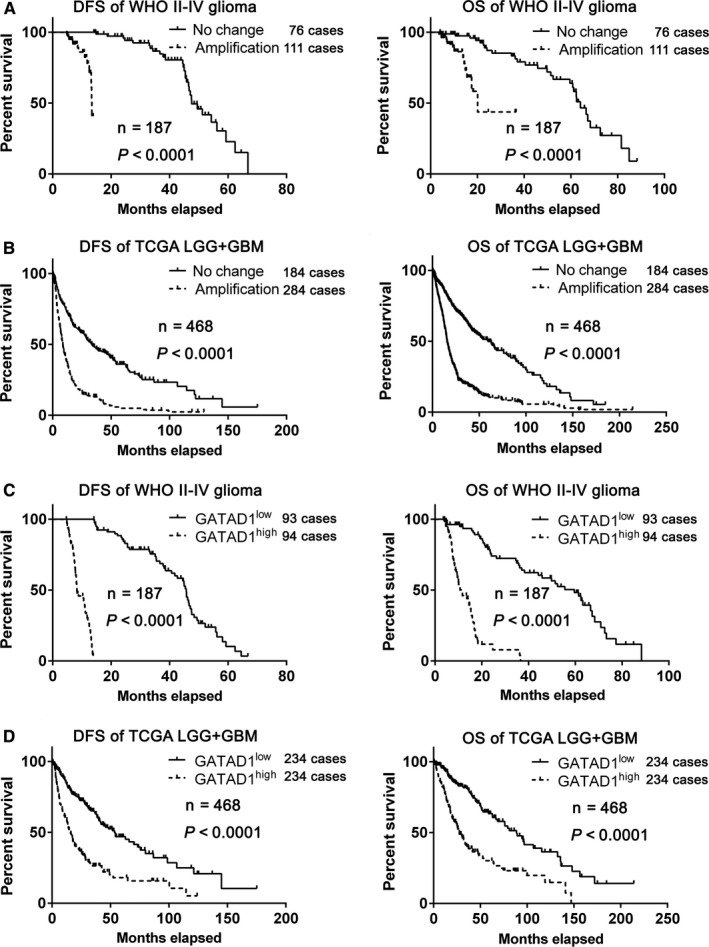
The amplification condition and expression level of GATAD1 indicated poor prognosis of glioma. (A) Kaplan‐Meier (KM) results of the survival condition of our WHO grades II~IV glioma patients samples stratified by GATAD1 gene amplification condition (DFS, left, OS, right, n = 187). (B) KM analysis of the survival condition of the LGG + GBM samples from TCGA database stratified by GATAD1 amplification condition (DFS, left, OS, right, n = 468). (C) KM results of the outcome of our WHO grades II ~ IV glioma samples stratified by GATAD1 LI (DFS, left, OS, right, n = 187). (D) KM results of the outcome of the LGG + GBM samples from TCGA database stratified by GATAD1 RNA level (DFS, left, OS, right, n = 468)

Kaplan‐Meier assay was performed to evaluate the effect of GATAD1 on glioma patients’ outcome. In our glioma samples (WHO grade II‐V) and TCGA glioma sample (LGG + GBM), the patients bearing GATAD1 gene amplification had shorter survival time (DFS and OS, Figure 2A,B). Furthermore, in our 187 glioma samples, the patients with higher GATAD1 IHC stating level had shorter survival times (median of GATAD1 LI = 31.2%, Figure 2C). The above trend was also verified in the LGG + GBM samples of TCGA dataset (median of log_2_ (TPM_GATAD1_ + 0.001) = 9.711, n = 468, Figure 2D). We found that GATAD1 amplification conditions also indicated shorter DFS and OS in wild‐type IDH1/2 or IDH mutation groups (Figure S1). Univariate and multivariate Cox regression of the survival data was also performed; the statistics results showed that both GATAD1 gene amplification and GATAD1 hyper‐expression were independent predictors of poorer survival of glioma patients (Tables S5‐S8).

### 3.3 GATAD1 induces the proliferation and malignancy of GBM cells

**Figure 3:**
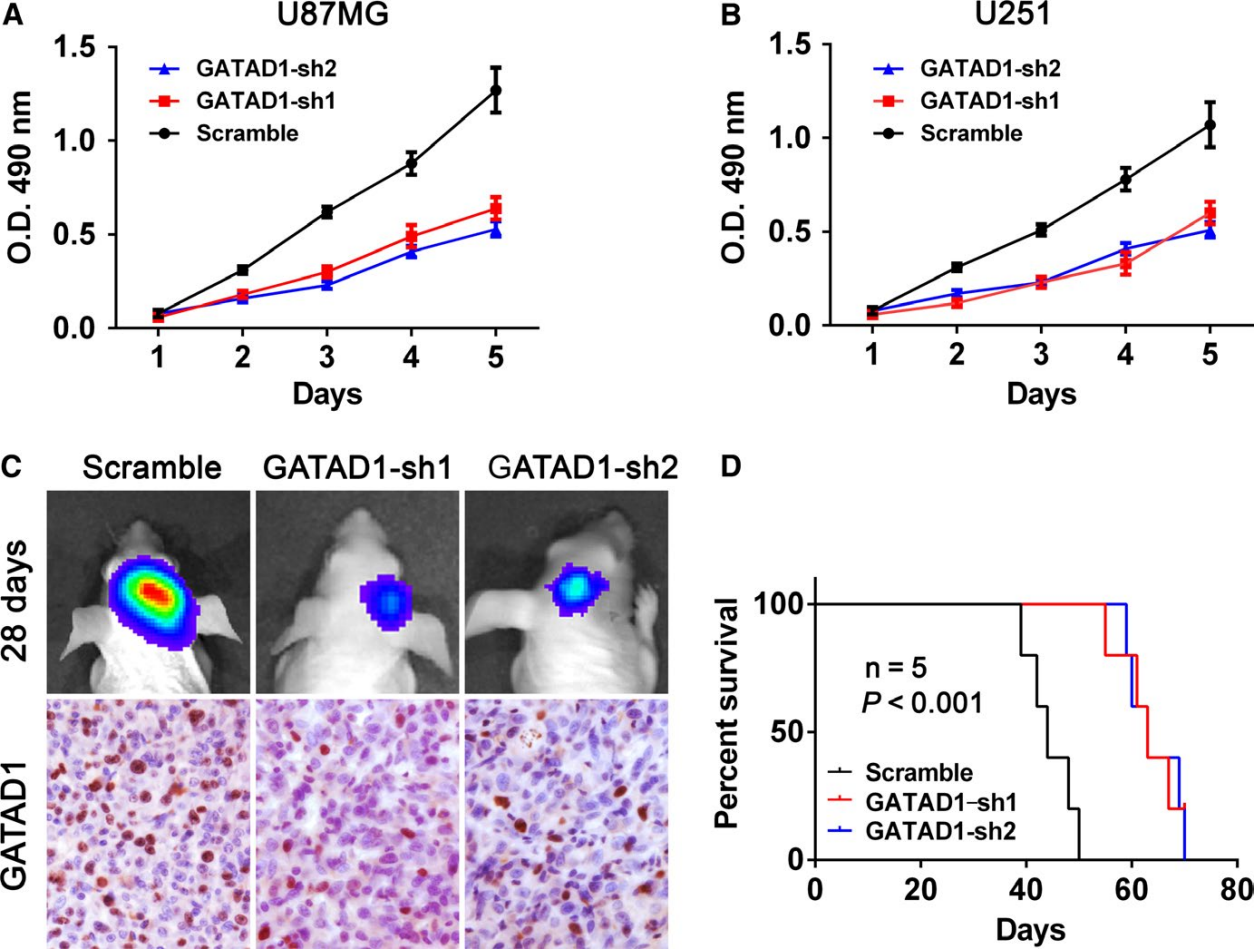
GATAD1 knockdown inhibits the proliferation of glioma cells. (A) MTT assays showed the proliferation condition among the U87MG cells of control (Scramble) and GATAD1‐knockdown (GATAD1‐sh1/sh2) groups. (B) The MTT proliferation assay among the U251 cells of control (Scramble) and GATAD1‐knockdown (GATAD1‐sh1/sh2) groups. (C) U87MG cells stable transfected control shRNA (Scramble) or GATAD1 shRNAs (GATAD1‐sh1/sh2) were transplanted into non‐obese diabetic severe combined immunodeficiency mice for 28 days (n = 5). Tumor bioluminescence image were taken using the ISVS system (upper). IHC staining was used to detect the GATAD1 level of the xenograft tumors from mice of the above groups (lower). (D) KM Survival results show that mice transplanted with GATAD1‐sh1/sh2 cells had better outcome than mice of the control group (Scramble, *P* < 0.001)

To evaluate the role of GTATAD1 on the proliferation and malignancy of GBM cells, we performed MTT proliferation and orthotopic tumor transplantation assays. The results indicated that knocking down of GATAD1 in GBM cells (GATAD1‐sh1/2) could significantly repress the proliferation of U87MG and U251 cells (Figure 3A,B). Then we evaluated the in vivo malignancy effect of GATAD1 in GBM. The U87MG cell expressing firefly luciferase were further stable transfected with scramble RNA (Scramble) or GATAD1 shRNA (GATAD1‐sh1/2). The aforementioned GBM cells were transplanted into NOD‐SCID mice (6‐week‐old, n = 5). The results showed that knocking down of GATAD1 could inhibit GBM tumor growth in vivo (Figure 3C, upper panel). The xenograft tumor samples were further detected using IHC for GATAD1 protein level; the results further confirmed that GATAD1 staining signals were weaker in the smaller GATAD1‐sh1/2 xenografts (Figure 3C, lower panel GATAD1). The KM results indicated that GATAD1 knockdown could prolong the survival time of xenografted mice (Figure 3D). The above results confirmed that GATAD1 could promote the proliferation and malignancy of GBM cells.

### 3.4 GATAD1 promotes the transcription of a series of genes related to proliferation and cell cycle of GBM cells

**Figure 4:**
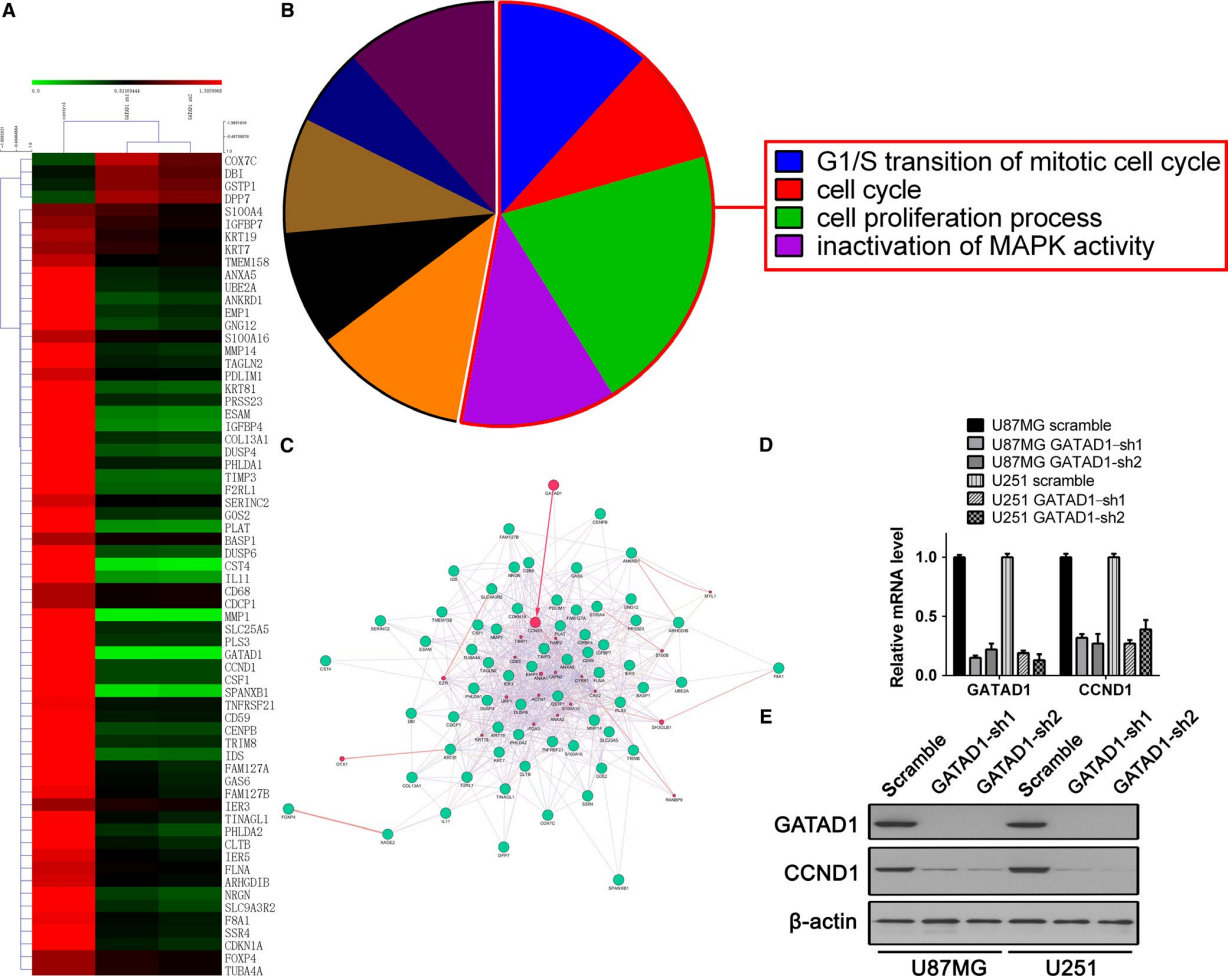
GATAD1 knockdown inhibits a series of genes involving proliferation and G1/S‐phase transition. (A) The cDNA microarray results of different expressed genes between control U87MG cells (Scramble) and GATAD1 knockdown cells (GATAD1‐sh1/sh2, n = 3 for each group). (B) The red box shows the most important pathways enriched from the GATAD1 targets gene. See also Table S10. (C) Network analysis results shows the relationships between GATAD1 and CCND1 (red circle). (D,E) The regulation of CCND1 by GATAD1 detected using microarray (A) was confirmed using qRT‐PCR (D) and Western blot (E)

To identify the downstream gene of GATAD1 tumor promoting function in GBM cells, we performed the cDNA microarray among scramble control U87MG cells (control) and GATAD1 knockdown U87MG cells (GATAD1‐sh1/2). Hierarchical Clustering results showed that 65 genes were enriched, four genes were upregulated and 61 genes were downregulated upon GATAD1 knockdown (data are shown as mean of each group, fold > 2, n = 3, FDR < 0.05, Figure 4A; Table S9). Among the 61 downregulated genes, the CCND1 gene expression was most correlated with GATAD1. The functional annotation results showed that the downregulated genes participated in the cell cycle G_1_/S transition and cell proliferation processes (Figure 4B; Table S9). The gene regulation network analysis result based on the Cytoscape GeneMANIA package showed that GATAD1 might directly regulate the transcription of CCND1 (Figure 4C). We then used qRT‐PCR and western blot to detect the expression level of GATAD1 and CCND1. The results confirmed that knocking down of GATAD1 would dramatically repress the expression of CCND1 in both U87MG and U251 cells (Figure 4D‐E).

### 3.5 GATAD1 enhances the transcription of CCND1 by binding to its promoter and inducing promoter histone acetylation

**Figure 5:**
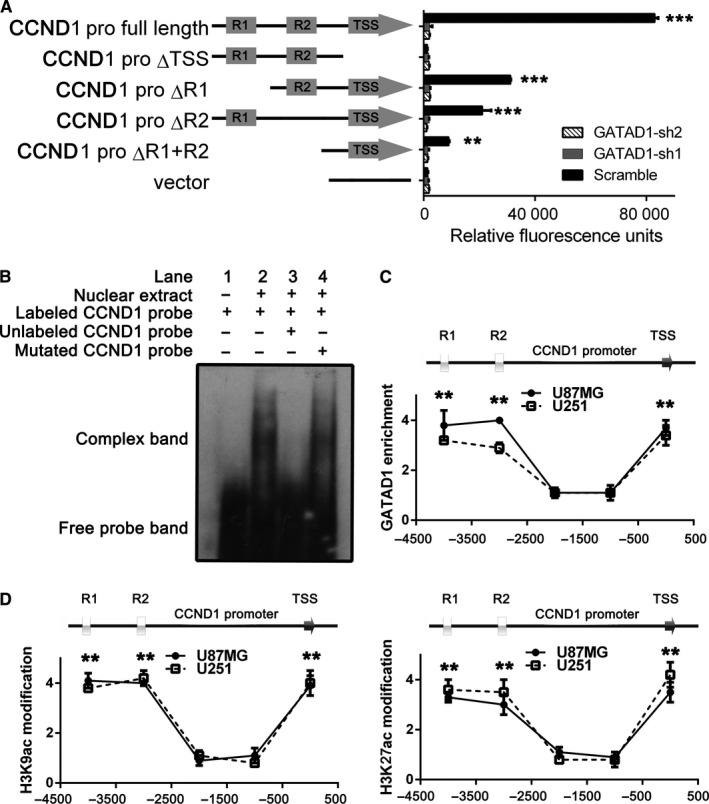
GATAD1 promotes CCND1 transcription by recognizing the conserved sequence of CCND1 promoter. (A) Luciferase assays were performed via the pGL3 vector containing the full‐length or different mutated CCND1 promoters. The pRL‐CMV luciferase vector was set as reference in U87MG with or without GATAD1 knockdown (Scramble or GATAD1‐sh1/sh2). (B) Electrophoretic mobility shift assay (EMSA) assay showed the interaction between GATAD1 protein and CCND1 promoter. (C) ChIP‐qPCR analyses results of the binding condition of GATAD1 with conserved regions on CCND1 promoter in U87MG and U251 cells. (D) ChIP‐qPCR results of the histone H3K9ac (left) and H3K27ac (right) modification of conserved regions on CCND1 promoter in U87MG and U251 cells. n = 3, **P < 0.01. The data are displayed as mean ± SD

GATAD1 could bind with the CCCNNCCC sequence of the chromatin. There are two conserved CCCNNCCC regions located on the promoter of CCND1 gene (R1 and R2); it is reasonable to presume GATAD1 could promote CCND1 gene transcription by recognizing the conserved regions. To confirm the hypothesis, we transfected a different luciferase reporter into U87MG cells which had high level GATAD1, the results showed that the transcription start site (TSS) region itself has basal transcription activity (CCND1 proΔR1 + R2). Several extensions of TSS core promoter including R1 and R2 could enhance the transcription (CCND1 proΔR1 and CCND1 proΔR2), the full‐length promoter had the highest transcription activity (CCND1 pro full length). The above data suggested that CCND1 enhanced CCND1 transcription by binding to R1, R2 and TSS sites. However, knocking down GATAD1 in U87MG cells repressed CCND1 transcription activity to basal levels (Figure 5A). We used EMSA to identify the binding affinity of GATAD1 to the conserved regions on CCND1 promoter. The purified GATAD1 protein could bind to labeled CCND1 probe (Figure 5B, lane 2), only the unlabeled completive probe would block the forming of shift band (Figure 5B, lane 3), the mutated probe would not be recognized by GATAD1 (Figure 5B, lane 4). ChIP‐qPCR results further confirmed that GATAD1 protein could interact with the R1, R2 and TSS sites of the CCND1 promoter (Figure 5C). During the transcription process, histone H3K9Ac and H3K27Ac modification often occured on certain conserved regions of the promoter. ChIP results show that the histone acetylation modifications were located at the same region as the GATAD1 binding sites (Figure 5D). The above results further indicated that GATAD1 could recognize histone acetylation regions of CCND1 promoter.

### 3.6 GATAD1 promotes the transcription of CCND1 by inducing long‐range chromatin conformation alternation

**Figure 6:**
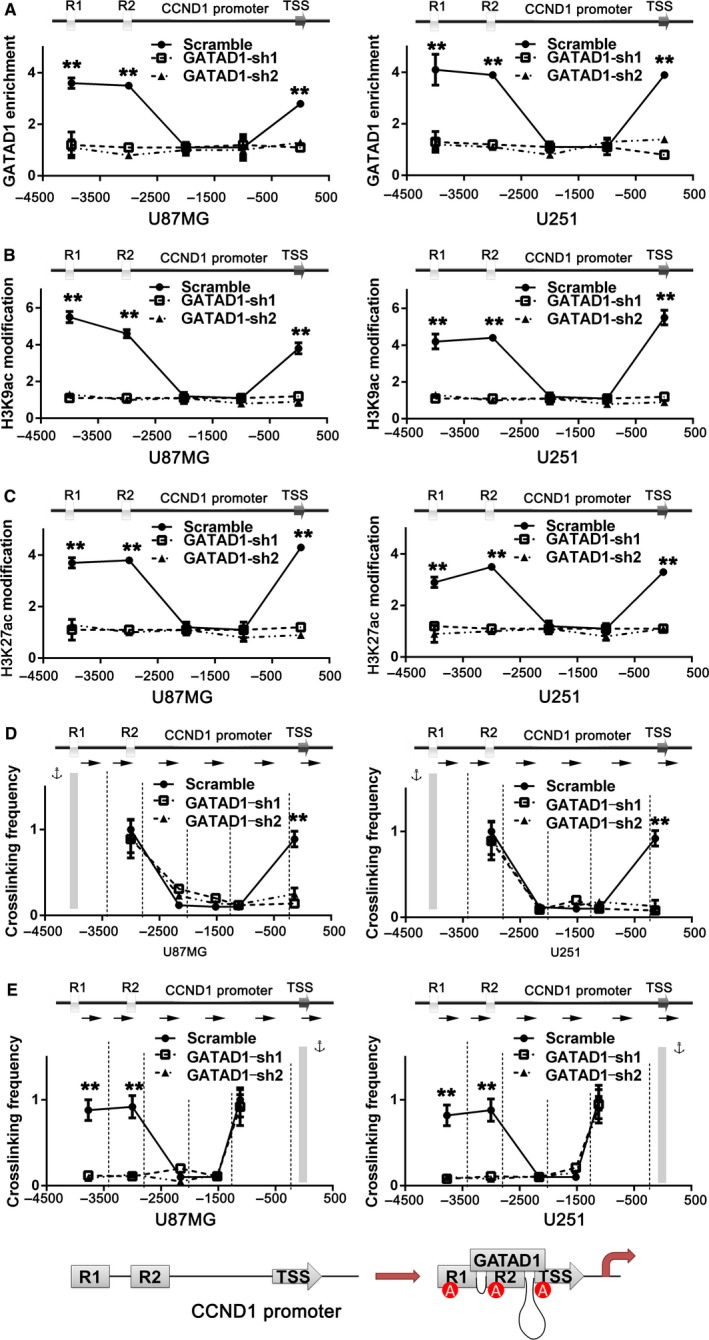
GATAD1 promotes CCND1 transcription via remodeling long‐range chromatin topology structure of the CCND1 prompter. (A) ChIP results show the enrichment condition of GATAD1 at CCND1 promoter after GATAD1 knockdown. (B,C) ChIP results show the histone H3K9ac (B) and H3K27ac (C) modification condition at CCND1 promoter after GATAD1 knockdown. (D) GATAD1 binding sites R1 was set as anchor, 3C result show the long‐range interaction among R1 to R2 and the TSS in GBM cells with or without GATAD1 knockdown. TaqI cutting sites were marked as vertical lines. The PCR primers are displayed as arrows. (E) TSS was set as anchor, 3C result of interaction among TSS to R1 and R2. (F) Schematic image presents the mechanism of GATAD1 inducing CCND1 transcription. n = 3, **P < 0.01. The data are displayed as mean ± SD

To further assess whether binding between GATAD1 and CCND1 promoter is essential for CCND1 promoter histone acetylation and CCND1 transcription activation, we knocking down GAGAD1 in U87MG and U251 cells. The ChIP result of GATAD1 showed that knocking down of GATAD1 would specifically inhibit the binding of GATAD1 to CCND1 promoter (Figure 6A). Then we used ChIP‐qPCR to detect the histone modification condition after GATAD1 knockdown, the result showed that, in both GBM cells, GATAD1 shRNA transfection would inhibit the histone H3K9Ac and H3K27Ac modification of GATAD1 binding sites on CCND1 promoter (Figure 6B,C). The above results indicated that GATAD1 specifically induced histone acetylation on its own binding sites of CCND1 promoter.

Histone acetylation is a hallmark event of transcription activation; the cis‐regulatory elements located far away from each other need chromatin topology structure alternation to initiate transcription. GATAD1 may induce the chromatin architectural alternation process. Chromosome conformation capture (3C) assays were performed to assess the architectural proximity among different conserved regions of CCND1 promoter. Genomic DNA was digested and religated with TaqI. In U87MG and U251 cells, we first set the R1 motif as PCR anchor and detected very strong association among R1, R2 and TSS, while the interactions were completely depleted after GATAD1 shRNA transfection (Figure 6D). We also set TSS as anchor to double check the above results, similar interactions between TSS and R1 and R2 were also detected (Figure 6E). These results further indicated that, by directly recognizing CCND1 promoter and promoting histone acetylation of the corresponding promoter regions, GATAD1 induced the long‐range chromatin conformation remodeling on CCND1 promoter and initiated gene transcription (Figure 6F).

### 3.7 GATAD1 promotes proliferation of GBM cells via CCND1 and by accelerating the cell cycle

**Figure 7:**
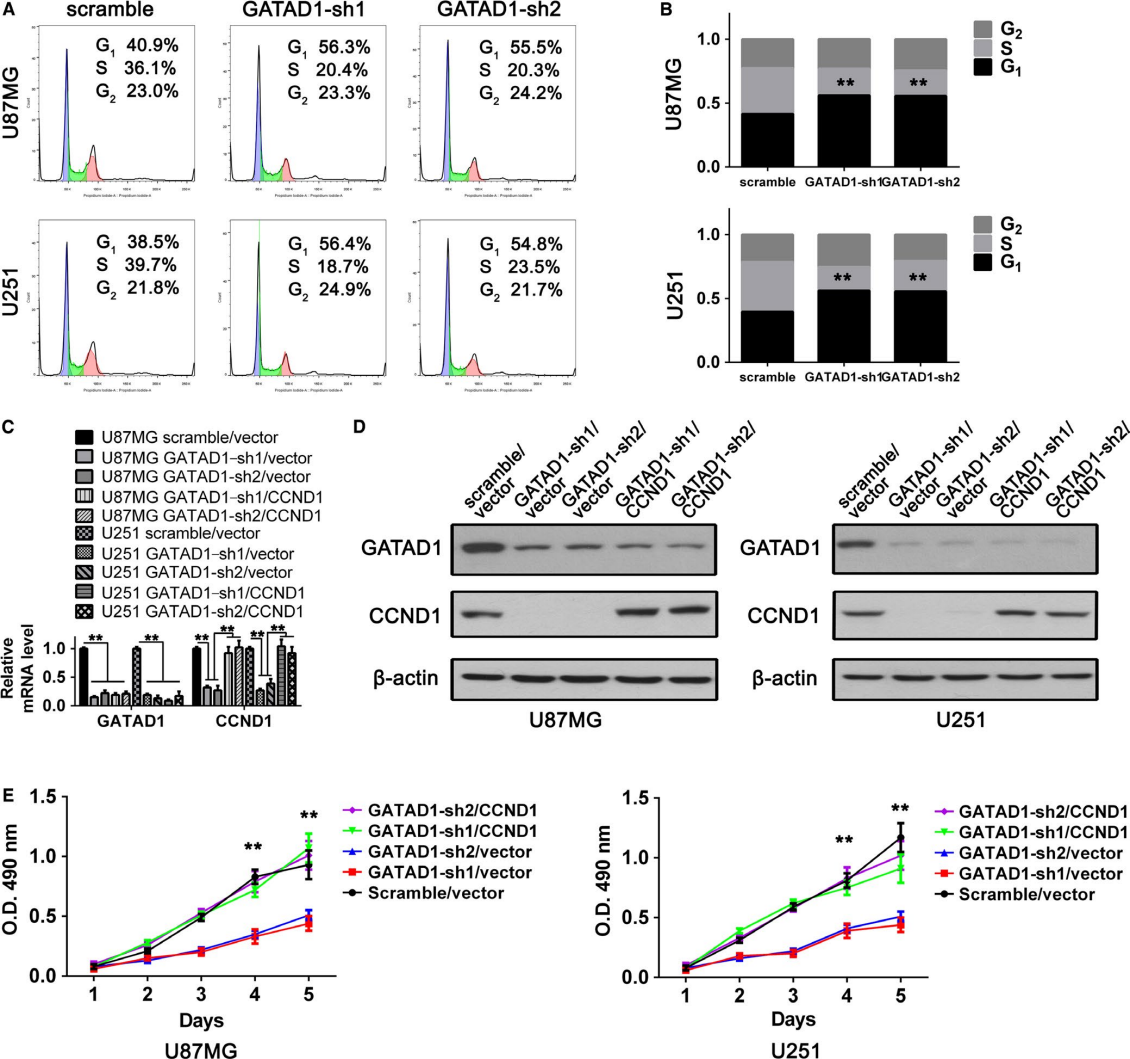
GATAD1 accelerates the GBM cell cycle, proliferation via CCND1. (A) Representative flow cytometry cell cycle analysis images of U87MG and U251 cells of the control (Scramble) and GATAD1‐knockdown (GATAD1‐sh1/sh2) groups. (B) Statistical analysis results of the cell cycle analysis of the aforementioned cells. (C) qRT‐PCR results of GATAD1 and CCND1 mRNA levels in U87MG and U251 cells of the Scramble/vector, GATAD1‐sh1/vector, GATAD1‐sh2/vector, GATAD1‐sh1/CCND1 and GATAD1‐sh2/CCND1 groups. (D) The protein level of GATAD1 and CCND1 in above cells detected using Western blot. (E) MTT assay results of the aforementioned cells. **P < 0.01

CCND1 is a key cell cycle G1/S checkpoint regulator. To identify the downstream mechanisms of GATAD1‐driven GBM cell proliferation, we transfected GATAD1 shRNAs into U87MG and U251 GBM cells via lentivirus. The flow cytometry results of GBM cells showed that knocking down of GATAD1 significantly inhibited cell cycle G1/S‐phase transition (Figure 7A,B). We then rehabilitated CCND1 expression in GATAD1 knocking down cells to confirm the importance of CCND1 to GATAD1‐promoted GBM proliferation. The above two GBM cells were stable‐infected by scramble control RNA/control vector (Scramble/vector), GATAD1‐shRNA/control vector (GATAD1‐sh1, sh2/vector) or GATAD1‐shRNA/CCND1 rescue expression (GATAD1‐sh1, sh2/ CCND1). The western blot and qRT‐PCR data confirmed that CCND1 expression was depleted in GATAD1 knockdown cells, the CCDN1 levels of rescue groups were recovered to basal levels in GBM cells (Figure 7C,D). After reestablish CCND1 expression by CCND1 rescue transfection (Figure 7C,D), the proliferation ability of GATAD1 knockdown cells was recover to nomal level (Figure 7E). Our results indicate a novel pathway by which GATAD1 gene copy amplification leads to GATAD1 overexpression; then the overexpressed GATAD1 promotes proliferation of GBM cells by targeting CCND1 and accelerating cell cycle G1/S‐phase transition.

## 4 DISCUSSION

In this study, we showed that GATAD1 gene copy number amplification frequently occurred in glioma tissues and GBM cell lines, this gene amplification leads to overexpression of GATAD1 gene in the tumor tissue compared to normal brain tissue. GATAD1 gene is located at 7q21,^20^ which is a documented potential mutational hotspot in glioma.^21^ Our results showed that GATAD1 was amplified in 56% of our glioma tissue and TGGA glioma database, GATAD1 amplification condition was dramatically correlated with its gene expression. These results suggested that GATAD1 overexpression in glioma is mostly induced by GATAD1 gene amplification. In our glioma samples and TCGA LGG + GBM database, the GATAD1 expression was upregulated in up to 65% of patients, which is slightly higher than the gene copy amplification rate. These results suggested that, compared to other genetic and epigenetic regulation mechanisms, gene amplification is the main reason for GATAD1 overexpression in glioma samples. IHC detection of GATAD1 protein expression in our 187 glioma patients’ tissue samples showed that GATAD1 was overexpressed in 69.2% of glioma patients. High GATAD1 level was dramatically correlated to high glioma grade. Moreover, GATAD1 gene amplification and GATAD1 overexpression were significantly correlated with poor survival and acts as independent indicator of shorter survival in both WHO grade II‐III LGG and grade IV GBM. GATAD1 gene amplification and overexpression could be a promising prognosis factor for all grades of glioma patients. The multivariate and univariate analysis results based on our glioma patients and the TCGA glioma database showed that GATAD1 is a novel glioma prognosis risk factor independent to WHO grades, age, IDH mutation condition and other clinical factors. Although some new molecular diagnosis markers such as IDH mutation had been included in the histopathology diagnostic criteria, great intragroup survival variations exist among individual patients, such as growth status and aggressiveness of tumors, still cause huge difficulty in predicting outcome of glioma patients. Some patients with low‐grade glioma still have a poor prognosis.^22^ As an additional prognostic predictor, GATAD1 is important to provide better prognosis assessment for glioma patient.

For now there is no publication regarding GATAD1 expression and function in gliomas. Previous results showed that GATAD1 could regulate cell cycle by targeting PRL3.^23^ We hereby found that GATAD1 was often overexpressed in glioma patients, the above results showed that GATAD1 may play an important role during the glioma oncogenesis process. The GATAD1 shRNA transfection significantly inhibited the proliferation of glioma cell. GATAD1 could bind to the DNA sequences via its N‐terminus zinc finger domain and promote gene transcription. As transcription factors, GATAD1 could control the expression of several downstream target genes, which might serve as a regulator of cell cycle and proliferation. We used cDNA microarray to screen the downstream gene of GATAD1 and found that CCND1 is the main target of GATAD1 in glioma cells.

In our present study, we found that there are several conserved CCCNNCCC sequences located on the TSS site and two upstream regions of the CCND1 promoter, GATAD1 could recognize the above sites and sequentially induce histone acetylation and long‐range chromatin conformation interaction among these conserved sites. GATAD1 and the recruited histone acetylated chromatin further induced the transcription of CCND1. Our results showed that GATAD1 is not only a transcription factor but also a novel chromatin architecture regulator.

CCND1, coupled with CDK4, acts as a key regulator of the cell cycle G1 phase transition checkpoint.^24^, ^25^ The transcription of CCND1 gene is controlled by different upstream pathways. The Wnt/β‐catenin pathway controls the CCND1 gene transcription in gastric cancer,^26^ colorectal carcinoma^27^ and liver cancer.^28^ In lung cancer,^29^ colon cancer^30^ and breast cancer,^31^ the EGF pathway could mediate the expression of CCND1. In this study, we found that GATAD1 could directly target the CCND1 gene, promoting CCND1 transcription and accelerating glioma cell G_1_–S cycle transition. The mechanism of GATAD1 amplification promoting glioma cell proliferation was mediated by CCND1.

In summary, we identified that GATAD1 gene copy amplification induced GATAD1 overexpression and played an oncogenetic role in patients with glioma. We also revealed a novel mechanism of glioma malignance driven by GATAD1 by sequentially inducing histone acetylation, chromatin conformation interaction and transcription of CCND1 gene (Figure 6F). Univariate and multivariate survival analysis showed that amplification and expression level of GATAD1 were independent predictors of poorer survival of glioma patients, which provided a strong rationale for focusing on GATAD1 in malignant glioma diagnosis and therapy.

## CONFLICT OF INTEREST

None declared.

## Data Availability

The data that support the findings of this study are openly available in [TCGA] at [https://www.cancer.gov/tcga], reference number.^32^
