# Supplementary figures and images for: GATAD1 gene amplification promotes glioma malignancy by directly regulating CCND1 transcription
Source: Cancer Med. 2019 Jul 8;8(11):5242–53. doi: 10.1002/cam4.2405 (PMC6718743; doi:10.1002/cam4.2405)

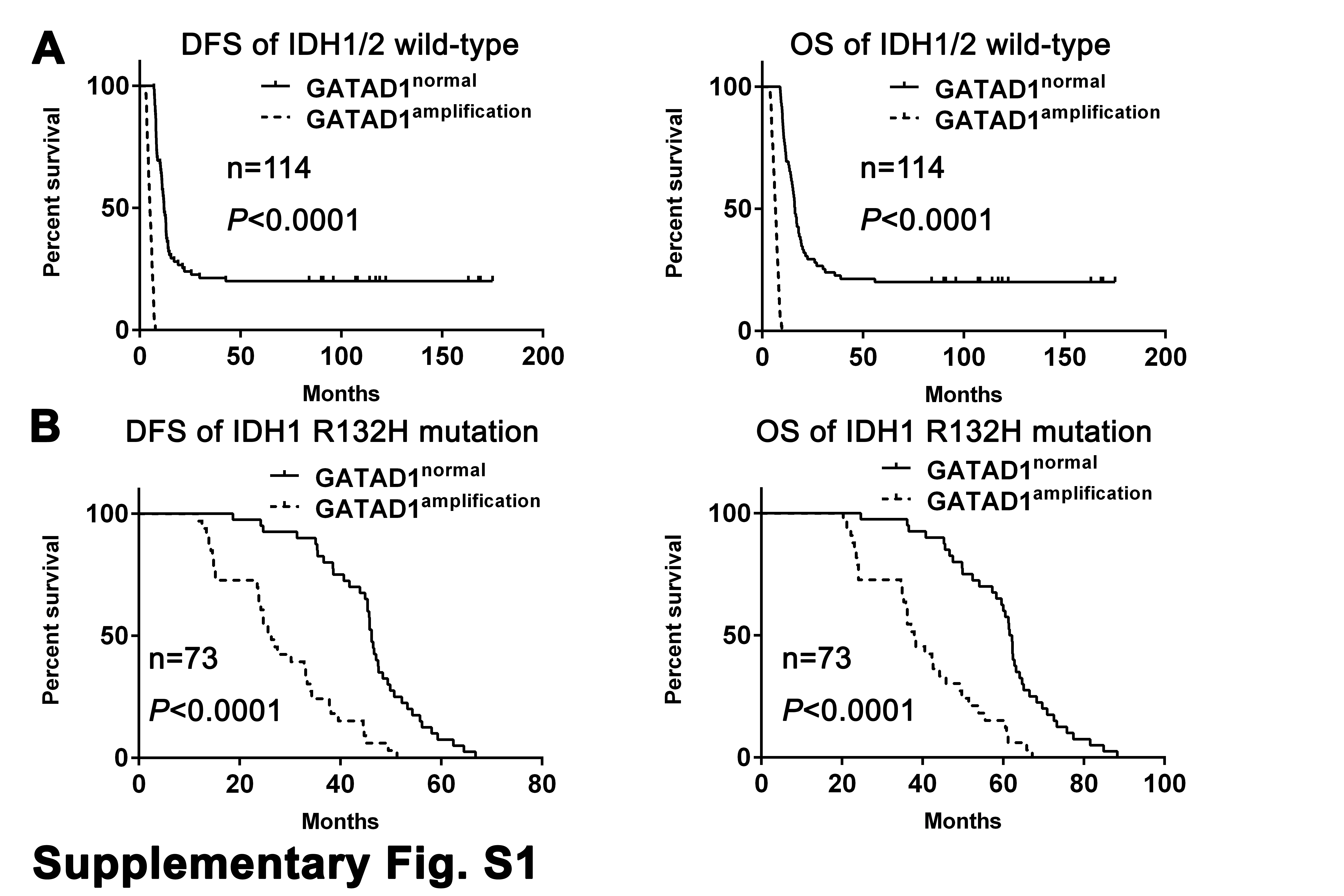

Supplement: Supplementary file 1 [file CAM4-8-5242-s001.tif]
