# Supplementary material for: GATAD1 gene amplification promotes glioma malignancy by directly regulating CCND1 transcription
Source: Cancer Med. 2019 Jul 8;8(11):5242–53. doi: 10.1002/cam4.2405 (PMC6718743; doi:10.1002/cam4.2405)
Supplement: Supplementary file 2 [file CAM4-8-5242-s002.doc]

**Supplementary Tables**

**Supplementary Table S1.** The pathological and clinical features of the glioma specimens

| Feature | Histological classification | | |
| --- | --- | --- | --- |
|  | Diffuse astrocytoma | Anaplastic astrocytoma | Glioblastoma |
|  | WHO Grade | | |
|  | II (n=40) | III (n=40) | IV (n=107) |
| IDH status | | | |
| Mutant type (IDH1 R132H) | 35 | 33 | 5 |
| Wild type (IDH1/2) | 5 | 7 | 102 |
| 1p19q status | | | |
| Codeletion | 0 | 0 | 0 |
| Normal | 40 | 40 | 107 |
| Gender | | | |
| Male | 22 | 21 | 57 |
| Female | 18 | 19 | 50 |
| Age (Year, Mean ± SD) | 43±11.9 | 47±13.9 | 56±12.4 |
| Predominant side | | | |
| Left | 18 | 18 | 58 |
| Right | 17 | 20 | 45 |
| Middle | 5 | 2 | 4 |
| Predominant location | | | |
| Frontal lobe | 31 | 26 | 50 |
| Temporal lobe | 5 | 8 | 41 |
| Parietal lobe | 1 | 4 | 8 |
| Occipital lobe | 1 | 1 | 6 |
| Pineal body | 0 | 0 | 0 |
| Saddle area | 0 | 0 | 0 |
| Thalamus | 0 | 0 | 0 |
| Cerebellum | 2 | 0 | 2 |
| Third ventricle | 0 | 1 | 0 |

Abbreviation: SD, standard deviation.

**Supplementary Table S2.** Primers used for promoter DNA ChIP-qPCR detection and probes for EMSA

| Primers |  | Sequence |
| --- | --- | --- |
| CCND1-promoter-1 | forward | 5’- TAAGGGGTCCGAATCCGAGT-3’ |
|  | reverse | 5’- GGCGCCTTAGAGTTCTGGTT-3’ |
| CCND1-promoter-2 | forward | 5’- CCAAAAGCAAGCAGTGTGGG-3’ |
|  | reverse | 5’- TTAAGCCCTTAAGTCGCCCG-3’ |
| CCND1-promoter-3 | forward | 5’- CCCCCATTGGCTCATGCTAA-3’ |
|  | reverse | 5’- CATAAAGAGGCTCGCCCACT-3’ |
| CCND1-promoter-4 | forward | 5’- GGAACCTTCGGTGGTCTTGT-3’ |
|  | reverse | 5’- AACCGGGAGAAACACACCTC-3’ |
| CCND1-promoter-5 | forward | 5’- CTGTGCTGCGAAGTGGAAAC-3’ |
|  | reverse | 5’- CAGGACCTCCTTCTGCACAC-3’ |
| CCND1-EMSA-probe | 5’-CCCCGGCCCCCCAAGCCCCCCGCGCCCCCTCCC-3’ | |
|  | 5’-GGGAGGGGGCGCGGGGGGCTTGGGGGGCCGGGG-3’ | |
| CCND1-EMSA-probe-mut | 5’-CGGCGGCGGCCCAAGCCGGCCGCGCCGGCTCCG-3’ | |
|  | 5’-GGAGCCGGCGCGGCCGGCTTGGGCCGCCGCCG-3’ | |

**Supplementary Table S3. Primers used for mRNA qRT-PCR detection**

| Primers |  | Sequence |
| --- | --- | --- |
| GATAD1 | forward | 5’-GGCCAGATCCCTTTCCCAG-3’ |
|  | reverse | 5’-GCGATAGGAAGAGCGGTGAG-3’ |
| CCND1 | forward | 5’-GCTGTGCATCTACACCGACA-3’ |
|  | reverse | 5’-TTGAGCTTGTTCACCAGGAG-3’ |
| GAPDH | forward | 5’-TGCACCACCAACTGCTTAGC-3’ |
|  | reverse | 5’-GGCATGGACTGTGGTCATGAG-3’ |

**Supplementary Table S4. Primers used for 3C**

| Primers |  | Sequence |
| --- | --- | --- |
| 3C-1 |  | 5’-CGAGCCGCCGCCGCAGGTTTC-3’ |
| 3C-2 |  | 5’-AAGCAGTGTGGGGGGAAGGGGA-3’ |
| 3C-3 |  | 5’-TCCCCGCCGGGCCCCAAATTCC-3’ |
| 3C-4 |  | 5’-AATCCTAATTCCCGGCGGGGGA-3’ |
| 3C-5 |  | 5’-GATCAGTACACTCGTTTGTTTAATT-3’ |
| 3C-6 |  | 5’-GCCTCAGGGATGGCTTTTGGGC-3’ |

**Supplementary Table S5.** Multivariate analysis for DFS and OS in patients with gliomas

| Factors | DFS | |  | OS | |
| --- | --- | --- | --- | --- | --- |
|  | HR(95%CI) | *P* |  | HR(95%CI) | *P* |
| **Gender** | 0.877(0.509-1.301) | 0.437 |  | 0.899(0.552-1.311) | 0.524 |
| **Age** | 1.166(1.113-1.207) | 0.012 |  | 1.201(1.099-1.311) | 0.042 |
| **Predominant side** | 0.921(0.332-1.124) | 0.301 |  | 0.903(0.372-1.220) | 0.405 |
| **Predominant location** | 0.992(0.811-1.204) | 0.190 |  | 1.170(0.705-1.211) | 0.331 |
| **WHO Grade** | 1.277(1.155-1.311) | <0.001 |  | 1.185(1.167-1.234) | <0.001 |
| **KPS** | 0.907(0.801-1.152) | 0.401 |  | 1.017(0.892-1.112) | 0.304 |
| **IDH status** | 0.335(0.192-0.371) | <0.001 |  | 0.304(0.257-0.327) | <0.001 |
| **GATAD1 LI** | 1.339(1.204-1.367) | <0.001 |  | 1.295(1.188-1.314) | <0.001 |
| **GATAD1 amplification** | 1.334(1.204-1.370) | <0.001 |  | 1.277(1.224-1.351) | <0.001 |

Abbreviations: HR, hazard ratio; CI, confidence interval; LI, labeling index.

**Supplementary Table S6.** Univariate analysis for DFS and OS in patients with gliomas

| Factors | DFS | |  | OS | |
| --- | --- | --- | --- | --- | --- |
|  | HR(95%CI) | *P* |  | HR(95%CI) | *P* |
| **Gender** | 0.821(0.677-1.201) | 0.4511 |  | 0.833(0.614-1.322) | 0.433 |
| **Age** | 1.271(1.144-1.297) | <0.001 |  | 1.271(1.188-1.292) | <0.001 |
| **Predominant side** | 0.901(0.811-1.221) | 0.351 |  | 0.903(0.792-1.301) | 0.331 |
| **Predominant location** | 1.110(1.033-1.214) | 0.041 |  | 1.103(1.021-1.280) | 0.033 |
| **WHO Grade** | 1.277(1.125-1.303) | <0.001 |  | 1.188(1.070-1.201) | <0.001 |
| **KPS** | 0.902(0.817-1.130) | 0.201 |  | 1.192(0.903-1.277) | 0.377 |
| **IDH status** | 0.133(0.059-0.141) | <0.001 |  | 0.117(0.086-0.120) | <0.001 |
| **GATAD1 LI** | 1.277(1.199-1.301) | <0.001 |  | 1.227(1.188-1.240) | <0.001 |
| **GATAD1 amplification** | 1.304(1.282-1.388) | <0.001 |  | 1.372(1.292-1.401) | <0.001 |

Abbreviations: HR, hazard ratio; CI, confidence interval; LI, labeling index.

**Supplementary Table S7.** Multivariate analysis for DFS and OS in TCGA GBM + LGG

| Factors | DFS | |  | OS | |
| --- | --- | --- | --- | --- | --- |
|  | HR(95%CI) | *P* |  | HR(95%CI) | *P* |
| **Gender** | 1.101(0.875-1.501) | 0.412 |  | 0.933(0.672-1.351) | 0.477 |
| **Age** | 1.254(1.210-1.367) | <0.001 |  | 1.244(1.192-1.334) | <0.001 |
| **Tumor location** | 0.911(0.783-1.401) | 0.351 |  | 1.075(0.788-1.2888) | 0.259 |
| **WHO Grade** | 1.222(1.192-1.312) | <0.001 |  | 1.203(1.181-1.288) | <0.001 |
| **KPS** | 0.82(0.601-1.301) | 0.402 |  | 1.021(0.733-1.501) | 0.511 |
| **IDH status** | 0.344(0.276-0.350) | <0.001 |  | 0.401(0.377-0.462) | <0.001 |
| **GATAD1 mRNA level** | 1.157(1.107-1.211) | 0.014 |  | 1.188(1.152-1.213) | 0.019 |
| **GATAD1 amplification** | 1.204(1.188-1.241) | <0.001 |  | 1.314(1.292-1.355) | <0.001 |

Abbreviations: HR, hazard ratio; CI, confidence interval.

**Supplementary Table S8.** Univariate analysis for DFS and OS in TCGA GBM+LGG

| Factors | DFS | |  | OS | |
| --- | --- | --- | --- | --- | --- |
|  | HR(95%CI) | *P* |  | HR(95%CI) | *P* |
| **Gender** | 0.901(0.834-1.214) | 0.355 |  | 1.167(0.892-1.388) | 0.324 |
| **Age** | 1.406(1.292-1.457) | <0.001 |  | 1.129(1.092-1.256) | <0.001 |
| **Tumor location** | 1.121(0.888-1.301) | 0.233 |  | 1.201(1.001-1.257) | 0.045 |
| **WHO Grade** | 1.331(1.122-1.377) | <0.001 |  | 1.214(1.177-1.341) | <0.001 |
| **KPS** | 0.882(0.599-0.992) | 0.091 |  | 0.811(0.692-0.903) | 0.033 |
| **IDH status** | 0.244(0.152-0.311) | <0.001 |  | 0.192(0.133-0.251) | <0.001 |
| **GATAD1 mRNA level** | 1.292(1.102-1.334) | 0.002 |  | 1.271(1.151-1.331) | <0.001 |
| **GATAD1 amplification** | 1.372(1.288-1.388) | <0.001 |  | 1.412(1.392-1.442) | <0.001 |

Abbreviations: HR, hazard ratio; CI, confidence interval.

**Supplementary Table S9.** Fold change of regulated genes in GATAD1-sh cells

| Gene name | U87MG (mean) | |
| --- | --- | --- |
|  | sh1 to control | sh2 to control |
| GATAD1 | 0.016745313 | 0.018123356 |
| CCND1 | 0.019030492 | 0.017844792 |
| CST4 | 0.030958799 | 0.018251829 |
| SPANXB1 | 0.036151178 | 0.053222567 |
| IL11 | 0.138729899 | 0.127691469 |
| PLAT | 0.15581334 | 0.146190351 |
| IGFBP4 | 0.168940237 | 0.152958319 |
| ESAM | 0.190752582 | 0.172356124 |
| IDS | 0.220696263 | 0.229639093 |
| TIMP3 | 0.233333333 | 0.227749577 |
| F2RL1 | 0.255335202 | 0.249730545 |
| KRT81 | 0.272577395 | 0.251144898 |
| DUSP4 | 0.281099867 | 0.270060309 |
| DUSP6 | 0.29586379 | 0.287216973 |
| ANKRD1 | 0.309594437 | 0.343901965 |
| CENPB | 0.316273421 | 0.323560028 |
| MMP1 | 0.325877052 | 0.324750563 |
| CSF1 | 0.326594701 | 0.326202159 |
| GNG12 | 0.333624964 | 0.373140857 |
| NRGN | 0.334146058 | 0.300127714 |
| PLS3 | 0.377464511 | 0.375788644 |
| PHLDA2 | 0.378225452 | 0.3213014 |
| SLC9A3R2 | 0.392868595 | 0.349002758 |
| G0S2 | 0.393655958 | 0.38732999 |
| EMP1 | 0.394503817 | 0.42259542 |
| COL13A1 | 0.396292656 | 0.383653981 |
| TRIM8 | 0.403388183 | 0.410331181 |
| SLC25A5 | 0.411608157 | 0.408887028 |
| MMP14 | 0.41810087 | 0.393580969 |
| PRSS23 | 0.420720522 | 0.406706288 |
| UBE2A | 0.421657382 | 0.452181987 |
| ANXA5 | 0.447551694 | 0.477134048 |
| CDKN1A | 0.456282031 | 0.419630377 |
| TAGLN2 | 0.464386824 | 0.447085786 |
| PHLDA1 | 0.465397826 | 0.46066783 |
| CLTB | 0.478253077 | 0.434878719 |
| CD59 | 0.479100605 | 0.485874892 |
| FAM127A | 0.498870091 | 0.44048128 |
| SSR4 | 0.511704609 | 0.480331429 |
| TNFRSF21 | 0.513739409 | 0.517632242 |
| TINAGL1 | 0.522662741 | 0.473972027 |
| GAS6 | 0.522833128 | 0.47202741 |
| F8A1 | 0.54016311 | 0.505753547 |
| FAM127B | 0.547343146 | 0.498903852 |
| IER5 | 0.569449296 | 0.535219467 |
| SERINC2 | 0.578466376 | 0.574044351 |
| ARHGDIB | 0.583431326 | 0.562323612 |
| PDLIM1 | 0.590676753 | 0.576671437 |
| TMEM158 | 0.609270639 | 0.637590168 |
| FLNA | 0.612937071 | 0.59051314 |
| S100A16 | 0.641343615 | 0.6274142 |
| CD68 | 0.657312979 | 0.666814468 |
| CDCP1 | 0.666222296 | 0.67622063 |
| BASP1 | 0.674177832 | 0.670262746 |
| KRT19 | 0.710974665 | 0.635662131 |
| IER3 | 0.726220378 | 0.698765971 |
| FOXP4 | 0.736645643 | 0.693654267 |
| TUBA4A | 0.737626714 | 0.697276883 |
| KRT7 | 0.76505417 | 0.684429327 |
| IGFBP7 | 0.782115137 | 0.723674926 |
| S100A4 | 0.884259074 | 0.742851414 |
| DBI | 1.570490265 | 1.398662913 |
| GSTP1 | 1.658633697 | 1.555322716 |
| DPP7 | 2.142015707 | 1.976767016 |
| COX7C | 2.300946955 | 1.880899222 |

**Supplementary Table S10.** Functional annotation of GATAD1 downstream genes from cDNA microarray data

| **Go term** | **Description** | **Enrich genes** | **Fold Enrichment** | ***P*-value** |
| --- | --- | --- | --- | --- |
| GO:0000086 | G1/S transition of mitotic cell cycle | CDKN1A, CCND1 | 20.3169994 | 9.52E-04 |
| GO:0010165 | cell cycle | CDKN1A, TUBA4A, PHLDA1 | 55.23684211 | 0.001272101 |
| GO:0016032 | cell proliferation process | KRT19, SLC25A5, KRT7, MMP1 | 4.532253711 | 0.003955468 |
| GO:0000188 | inactivation of MAPK activity | DUSP4, DUSP6 | 11.44064044 | 0.004908624 |
| GO:0001958 | endochondral ossification | COL13A1, MMP14 | 9.658901352 | 0.007838217 |
| GO:0006508 | proteolysis | PLAT, MMP14, PRSS23, DPP7, MMP1 | 18.80403135 | 0.010726505 |
| GO:0010033 | response to organic substance | ANXA5, TIMP3 | 18.80403135 | 0.010726505 |
| GO:2000352 | negative regulation of endothelial cell apoptotic process | GAS6, IL11 | 147.2982456 | 0.013274283 |
| GO:0045214 | sarcomere organization | KRT19, ANKRD1 | 7.319167484 | 0.016547921 |

**Supplementary Table S11.** Antibody information

| **Antibody** | **Application** | **Dilution** | **Supplier** | **Cat. No.** |
| --- | --- | --- | --- | --- |
| **GATAD1** | **IHC**  **IB**  **ChIP** | IHC, 1:75  IB, 1:1000  8 μg/ml | Santa Cruz [Dallas, TX] | sc-81092 |
| **CCND1** | **IB** | IB, 1:1000 | Cell Signaling Technology [Danvers, MA] | #2978 |
| **H3K9Ac** | **ChIP** | 8 μg/ml | Abcam [Cambridge, UK] | ab10812 |
| **H3K27Ac** | **ChIP** | 8 μg/ml | Abcam [Cambridge, UK] | ab4729 |
| **β-actin** | **IB** | IB, 1:20000 | Abcam [Cambridge, UK] | ab8226 |
| **Normal Rabbit IgG** | **ChIP** | ChIP, 8 μg/ml | EMD Millipore [Billerica, MA] | 12-370 |
| **Normal Moure IgG** | **ChIP** | ChIP, 8 μg/ml | EMD Millipore [Billerica, MA] | 12-371 |
| **Anti-Rabbit IgG HRP** | **IB** | IB, 1:5000 | Abcam [Cambridge, UK] | ab6721 |
| **Anti-Mouse IgG HRP** | **IB** | IB, 1:5000 | Abcam [Cambridge, UK] | ab6789 |

**Supplementary Figure Legends**

**Supplementary Figure S1. GATAD1 amplification correlate with poor patients’ prognosis in IDH wild-type and mutation patients. (A)** Survival analysis of the relation between GATAD1 amplification condition and patients’ outcome for all wild-type IDH1/2 glioma (DFS, left; OS, right, n=114). **(B)** Survival data of GATAD1 amplification and patients bearing IDH1 R132H mutation (DFS, left; OS, right, n=73). Patients were stratified by GATAD1 amplification condition.
